# Intrinsic connectome organization across temporal scales: New insights from cross-modal approaches

**DOI:** 10.1162/netn_a_00114

**Published:** 2020-02-01

**Authors:** Sepideh Sadaghiani, Jonathan Wirsich

**Affiliations:** Psychology Department, University of Illinois at Urbana-Champaign, Urbana, IL, USA; Beckman Institute for Advanced Science and Technology, University of Illinois at Urbana-Champaign, Urbana, IL, USA; Beckman Institute for Advanced Science and Technology, University of Illinois at Urbana-Champaign, Urbana, IL, USA

**Keywords:** Connectome, Intrinsic, Multimodal, EEG, MEG, fMRI

## Abstract

The discovery of a stable, whole-brain functional connectivity organization that is largely independent of external events has drastically extended our view of human brain function. However, this discovery has been primarily based on functional magnetic resonance imaging (fMRI). The role of this whole-brain organization in fast oscillation-based connectivity as measured, for example, by electroencephalography (EEG) and magnetoencephalography (MEG) is only beginning to emerge. Here, we review studies of intrinsic connectivity and its whole-brain organization in EEG, MEG, and intracranial electrophysiology with a particular focus on direct comparisons to connectome studies in fMRI. Synthesizing this literature, we conclude that irrespective of temporal scale over four orders of magnitude, intrinsic neurophysiological connectivity shows spatial similarity to the connectivity organization commonly observed in fMRI. A shared structural connectivity basis and cross-frequency coupling are possible mechanisms contributing to this similarity. Acknowledging that a stable whole-brain organization governs long-range coupling across all timescales of neural processing motivates researchers to take “baseline” intrinsic connectivity into account when investigating brain-behavior associations, and further encourages more widespread exploration of functional connectomics approaches beyond fMRI by using EEG and MEG modalities.

## Introduction

Although even the simplest behaviors and conscious percepts involve a distributed set of brain regions, new empirical observations continue to challenge our understanding of such large-scale neural connectivity. Until about the mid-2000s, cognitive neuroimaging studies using functional magnetic resonance imaging (fMRI) almost exclusively focused on the brain’s response to experimentally controlled events. The discovery of a stable intrinsic functional connectivity (FC) organization (Beckmann et al., 2005; Biswal et al., 1995; Greicius et al., 2003) has drastically extended the focus of human functional neuroimaging (Raichle, 2009). The investigation of this stable organization was later expanded to whole-brain functional graphs or “connectomes” (Achard et al., 2006), building on the notion of a structural connectome (Sporns et al., 2005). This discovery revealed that the larger proportion of neural activity is continuously ongoing irrespective of specific external events and cognitive challenges (hence “intrinsic”), and is governed by FC across large-scale neurocognitive networks both during task-free resting state and various tasks (Cole et al., 2014; Krienen et al., 2014).

**Figure 1.**
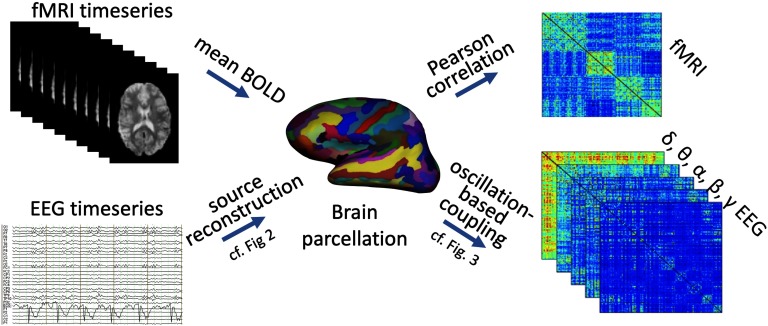
Whole-brain connectomes can be derived for various functional modalities covering different temporal scales. (Top) fMRI BOLD signal time courses are averaged across all voxels falling within each brain region of a whole-brain parcellation, such as an anatomically or functionally defined parcellation atlas. Functional connectivity is then derived as the pairwise dependence of signal fluctuations for each pair of brain regions of the atlas, most commonly by using Pearson correlations. The ensuing connectivity matrix of all-by-all brain regions reflects the fMRI-derived whole-brain functional connectome. (Bottom) EEG (or MEG) sensor space signal time courses are source reconstructed onto a whole-brain parcellation (cf. Figure 2). This process can be performed for various different frequency bands, often comprising canonical oscillation bands *δ* through *γ*. Oscillation-based FC is then defined for each pair of brain regions, typically by using either phase coupling or amplitude coupling (cf. Figure 3). The ensuing FC matrix of all-by-all brain regions (for each band) reflects the neurophysiologically derived whole-brain functional connectome. Note that using the same parcellation for different data modalities permits direct comparison of the respective connectomes.

Here, we emphasize another major conceptual advance that is currently emerging through direct comparison of whole-brain FC across data modalities that operate at different timescales (Figure 1). Moving beyond fMRI, this advance suggests that a spatially similar connectome organization governs long-range neural FC across connectivity measures and timescales. A comparable intrinsic large-scale network organization has been observed for infraslow fluctuations around 0.1 Hz (fMRI: Beckmann et al., 2005; EEG recorded concurrently to fMRI: Hiltunen et al., 2014) and for the full breadth of canonical oscillations up to the high *γ* frequency range around 100 Hz (Deligianni et al., 2014; Finger et al., 2016; Hipp & Siegel, 2015; Tewarie et al., 2018; Wirsich et al., 2017b).

The central goal of this review is to provide an overview of comparative approaches to intrinsic whole-brain connectome organization across data modalities. For the purpose of this review we collectively refer to various electrophysiological and electromagnetic recordings as neurophysiological methods. These methods comprise magnetoencephalography (MEG), electroencephalography (EEG), and intracranial recordings in animals and humans electrocorticography (ECoG). At the cost of limited spatial resolution or coverage, these data modalities provide real-time temporal information that contrast the less direct and temporally blurred measures of neural activity based on fMRI. Whereas the first investigations of whole-brain connectivity organization in functional and structural MRI modalities date back to the mid-2000s (Beckmann et al., 2005; Hagmann, 2005; Sporns et al., 2005), such investigations in neurophysiological data are more recent (Brookes et al., 2011; Hillebrand et al., 2012; Hipp et al., 2012). This delay is largely due to the methodological challenges of neurophysiological data modalities with extensive cortical coverage, that is, MEG and EEG, for which solutions have been developed (Hassan & Wendling, 2018; O’Neill et al., 2017) (cf. Box 3).

The review begins by briefly introducing what is known about the functional connectome from the fMRI literature (section 2). We then discuss the more recent advances in neurophysiological whole-brain FC and the degree to which they converge with the fMRI-based (section 3) and structural MRI-based connectome organization (section 4). Sections 3 and 4 will also highlight studies in concurrently recorded EEG-fMRI. Unless otherwise stated, we will focus on task-free resting-state studies, as most investigations of the intrinsic connectome have been undertaken at rest. We will close by discussing possible neurobiological scenarios that may explain the broad range of timescales governed by a universal connectome organization, and the implications for our understanding of long-range communication in the brain.

## 2. FUNCTIONAL CONNECTOMES BASED ON THE HEMODYNAMIC SIGNAL

At the turn of the millennium, about a decade after the birth of fMRI, functional neuroimaging began to substantially extend beyond investigations of task-related activation and FC changes among small sets of task-relevant brain regions. This paradigm shift toward whole-brain FC approaches first appeared in task-free resting-state studies and gradually extended to task settings. As fMRI-based connectomics has been extensively covered in prior literature (e.g., Buckner et al., 2013; Smith et al., 2013), the following section provides only a brief introduction. For a brief overview of core methodological considerations see Box 2.

### 2a. Discovery of Intrinsic Connectivity Networks

Only 3 years after the first human blood oxygen level dependent (BOLD) signal recordings, it was discovered that spontaneous (i.e., task-independent) BOLD signal fluctuations are temporally correlated across distant brain areas. This discovery initially comprised the somatomotor (SM) network (Biswal et al., 1995) and later the default mode network (DMN) (Greicius et al., 2003). It soon became evident that the observation applied to all major neurocognitive systems from sensory to higher order control systems, leading to the notion of intrinsic connectivity networks (ICNs) (Damoiseaux et al., 2006; De Luca et al., 2006; Fox et al., 2005).

### 2b. Whole-Brain Connectivity Graphs

Initially driven by the fundamental goal to mathematically describe the emergence of conscious awareness (Edelman, 1990; Edelman & Tononi, 2001), Tononi, Sporns and Edelman introduced an information-theoretic concept of whole-brain segregation and integration (Tononi et al., 1994). Later, Sporns and (independently) Hagman coined the term “connectome” for a whole-brain graph representation of anatomical connectivity based on fiber tracking of noninvasive diffusion MRI (dMRI) (Hagmann, 2005; Sporns et al., 2005). This advance coincided with the above-described discovery of a stable ICN architecture.

Consequently, whole-brain connectivity approaches were rapidly adopted to fMRI-derived “functional connectomes.” In functional connectomes, the strongest dependencies emerge across regions of the above-described ICNs (Figure 1, top row). Graph representations opened the functional neuroimaging field to complex network tools well developed in other sciences (Rubinov & Sporns, 2010). Functional connectome characteristics inform about differences in behavior within subjects (Sadaghiani et al., 2015), across healthy individuals (Finn et al., 2015; Nomi et al., 2017), and between clinical populations (Fornito & ullmore, 2010).

It is important to note that the spatial organization of the fMRI-derived connectome and its ICNs is very stable, experiencing only minor task-related changes in FC (Cole et al., 2014; Gratton et al., 2018; Krienen et al., 2014). Despite some degree of change, this organization largely persists in the absence of consciousness such as during sleep or anesthesia (Amico et al., 2017; Hutchison et al., 2013; Picchioni et al., 2013; Wirsich et al., 2017a). The collective spatial organization over all ICNs is thus considered the brain’s intrinsic functional architecture (Petersen & Sporns, 2015). The functional raison-d’$\hat{\text{e}}$tre of a relatively stable connectivity pattern that comes at a high energy cost may be provided by predictive coding accounts. Such accounts view this architecture as a “memory system” modeling the statistical structure of the world (Sadaghiani et al., 2010a; Sadaghiani & Kleinschmidt, 2013).

### 2c. Dynamic Connectivity Reconfigurations

Beyond the above-described stability of the fMRI-derived FC organization, reconfigurations are observed when the connectome is constructed from shorter periods (“dynamic” or “time-varying” connectivity) rather than the full recording (“static” or “time-averaged” connectivity) (Allen et al., 2014; Chang & Glover, 2010; Griffa et al., 2017; Liu & Duyn, 2013; Tagliazucchi et al., 2012b; Vidaurre et al., 2017). Considerable methodological debates notwithstanding (Hindriks et al., 2016; Laumann et al., 2017), the interest in dynamic FC has grown tremendously over the last few years (Cohen, 2017; Keilholz et al., 2017; Preti et al., 2017). The core driver behind this interest is the fact that cognition is inherently dynamic. Therefore, FC reconfigurations are likely to be associated with cognitive processes, and by extension, differences in cognitive abilities across individuals and clinical populations. This association of at least portions of observed FC dynamics with cognitive processes finds support in their impact on trial-by-trial perceptual outcomes (Sadaghiani et al., 2015), and in the cross-subject similarity of FC dynamics while listening to an identical story (stimulus-induced intersubject correlations; Simony et al., 2016). The neurophysiological counterparts of fMRI FC dynamics are discussed at the end of section 3c.

## 3. FUNCTIONAL CONNECTOMES BASED ON NEUROPHYSIOLOGICAL SIGNALS AND THEIR RELATIONSHIP TO FMRI-BASED CONNECTOMES

**Figure 2.**
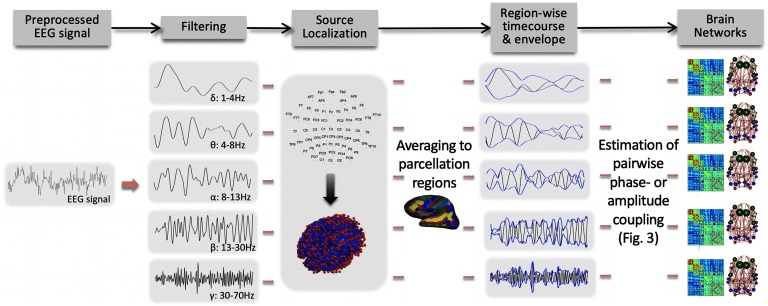
An example pipeline for constructing whole-brain connectomes from neurophysiological signals. In this example, after preprocessing, scalp EEG or MEG data undergo filtering to frequency bands of interest, source localization to regions of a brain parcellation, and estimation of connectivity across region pairs. Although all these steps are typically considered important, they can occur in different orders and by using different algorithms. Source localization is crucial because the mapping of EEG/MEG sensor data to brain regions is not linear, and several sensors can detect the activity of the same neuronal source (Farahibozorg et al., 2018). Indeed, empirical comparisons show that the spatial topology of FC differs heavily between source and sensor space (Lai et al., 2018). Beyond source localization, another important step to avoid source leakage and false positives in FC is to exclude any relationship at zero lag between two signal time courses, as leakage is assumed to propagate instantaneously (Palva et al., 2018; Palva & Palva, 2012). Various methods are available to this end (Brookes et al., 2011; Nolte et al., 2004; Stam et al., 2007). As further detailed in Figure 3, the connectivity measure of interest can be based either on phase (e.g., Wirsich et al., 2017b) or amplitude of the oscillatory signal (e.g., Deligianni et al., 2014). Collectively, these steps result in a connectivity matrix for each oscillation band, which may be interrogated as a graph (right column). Illustration modified with permission from Deligianni et al. (2014).

Compared with fMRI-based functional connectomes, estimating whole-brain FC patterns from neurophysiological signals is a more recent development. There is a long-standing view that oscillatory neural activity and its synchronization across brain regions facilitates long-range communication. Yet, such communication has traditionally been evaluated across a small set of sensors or reconstructed sourcesand temporally confined to relatively brief task-related processes (Singer, 1999; Uhlhaas et al., 2009; Varela et al., 2001). There is a well-established understanding that even in the absence of task, neurophysiological signal power organizes in reoccurring coarse spatial patterns (“microstates”) (Koukkou-Lehmann et al., 1980; Lehmann et al., 1987). However, deriving neurophysiological whole-brain connectomes is a relatively recent concept (Figure 2) (e.g., Deligianni et al., 2014; Hipp & Siegel, 2015; Tewarie et al., 2016). The current section discusses this latter view of ongoing oscillatory processes as functional dependencies unfolding continuously across distributed networks governing the whole brain.

### 3a. Conceptual Considerations

**Figure 3.**
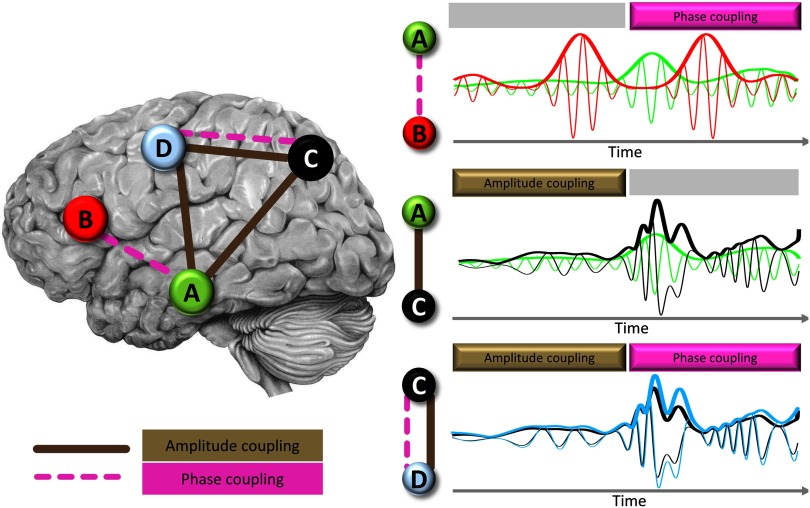
Schematic view of phase coupling and amplitude coupling as two different neurophysiological mechanisms of functional connectivity. (Left panel) Graph view of FC organization between four nodes and their connecting edges depicting either phase coupling (dashed pink lines) or amplitude coupling (solid brown lines). (Right panel) Narrow lines show time courses of neural activity in a given oscillatory frequency band overlaid for the two nodes involved in a given connection. Thicker lines in the plots show amplitude envelope of the signals with corresponding colors. Colors of nodes (left panel) and time courses (right panel) correspond to each other. The top, middle, and bottom right plots show connections with phase coupling only (A and B), amplitude coupling only (A and C), or both types of coupling (C and D), respectively.

Despite methodological challenges, first and foremost source leakage (see Box 3; Palva et al., 2018), connectomes can be successfully derived from EEG/MEG (as evidenced by the findings detailed in sections 3b, 3c, and 4). Neurophysiological connectomes can be constructed based on amplitude coupling or phase coupling (Figure 3), and further assessed across the full frequency range of neural population activity (infraslow to *γ* band). The rich information of neurophysiological signals thus provides multiple concurrent connectomes (Figures 1 and 2). Should we expect the neurophysiological connectomes at different temporal scales to differ from each other and from the fMRI-derived connectome? In our opinion, the prior literature implicitly reflects two conceptual viewpoints that lead to divergent predictions in regard to cross-scale and cross-modality correspondence of FC organization.

The first viewpoint predicts that FC organization is sensitive to timescales. This view arises on the basis of task-based neurophysiological experiments showing that both local power and long-range coupling in different canonical oscillation bands have distinct functional roles. Both local power and coupling are consequently more strongly tied to brain areas involved in the respective cognitive functions. For example, both amplitude (especially pronounced in occipito-parietal areas; Gould et al., 2011; Haegens et al., 2011) and phase coupling of the *α*-rhythm (Doesburg et al., 2009; Palva & Palva, 2007) are linked to selective prioritization of specific processes and the allocation of attention, especially in the visuo-spatial domain (Sadaghiani & Kleinschmidt, 2016). Another example of frequency-specific functional specialization is the role of the *θ* rhythm in navigation, memory encoding, and retrieval, especially pronounced in the hippocampus but also relevant neocortex (local amplitude: Buzsáki, 2005; Klimesch et al., 1996; amplitude coupling: Ekstrom et al., 2005; phase coupling: Backus et al., 2016). Conversely, *γ*-oscillations are thought to generally reflect local representations of item content (e.g., individual stimuli; Jacobs & Kahana, 2009). Their amplitude and long-range coupling are thus more widely observed in/across respective content-specific cortical areas (Fries, 2009; Rohenkohl et al., 2018). In summary, oscillation amplitudes (Kahana, 2006), oscillation phase cycle (VanRullen, 2016), and, importantly, oscillation-based FC (Palva & Palva, 2018) correlate with behavioral outcomes on different cognitive processes in a frequency-dependent manner.

This synthesis of prior literature (rather than any individual study) may lead to the prediction that the spatial organization of oscillation-based FC differs heavily across frequencies, such that certain region pairs primarily couple in particular frequency bands. Specifically, a connectome based on *α*-oscillations is likely not reflecting coupling of the same functional content as a connectome based on *γ*-oscillations. This functional specialization of each frequency band suggests that FC in that band would primarily occur across brain regions involved in the respective function. Important for our discussion, this frequency specificity of FC distribution would imply that the spatial topography differs substantially across connectomes derived from different frequency bands. By extension, the spatial relationship across electrophysiological and fMRI-derived connectomes would differ across different oscillation frequency bands.

Another viewpoint in favor of a timescale-invariant spatial organization emerges from computational and modeling studies. In an early example, Honey et al. (2007) simulated neuronal activity on different timescales by using a neural mass model (Larter et al., 1999; Morris & Lecar, 1981). They showed that synchronization levels derived from faster timescales (10 Hz) correlate with the infraslow fluctuations of the simulated BOLD signal (using the Balloon-Windkessel model from Friston et al. (2003)). Deco et al. (2009) demonstrated that using realistic time delays and coupling strengths in a neuronal model (Wilson-Cowan Oscillators) leads to oscillators synchronous at 40 Hz (*γ*), which in turn exhibit amplitude fluctuations in the infraslow range of the BOLD signal <0.01 Hz. Importantly, an ICN organization at a slow timescale did not only emerge in this specific model but has similarly been observed in other modeling approaches (FitzHugh-Nagumo oscillators; Ghosh et al., 2008), chaotic fluctuations (Honey et al., 2007), and a reduced Wong-Wang model (Deco et al., 2013; Hansen et al., 2015). Beyond the above-described models that fit fMRI from dMRI data, Schirner et al. (2018) have recently shown that it is possible to fine-tune the fit between empirical and simulated fMRI (from dMRI using a Wong-Wang model) by injecting concurrently recorded source-reconstructed EEG power. Cabral et al. (2014) demonstrated that FC modeled from structural connectivity using a Kuramoto model of phase-coupled oscillators is related to empirical MEG FC (envelope correlation) across all frequency bands. These observations suggest that neurophysiological recordings either hold supplementary information to model the structure-function relationship or help to improve SNR of the functional measures by providing a second independent measure of FC.

In summary, the above-described models predict the emergence of an intrinsic FC organization as a result of cluster synchronization between nodes at faster timescales (for review see Breakspear, 2017; Deco et al., 2011). According to these models, the function-structure coupling is strong across long timescales, whereas dynamic subnetwork configurations arise from shorter timescales (Deco et al., 2011). Collectively, the discussed models suggest a common spatial organization across all timescales when averaged over sufficiently long periods.

However, the presented models assume homogenous circuit properties of the underlying model. Conversely, it has recently been shown that relaxing those parameters (such as allowing for recurrent connection strength and excitatory subcortical input to differ across cortical regions) can improve the fit to empirical fMRI (Wang et al., 2019). While—as discussed above—the global view puts forward a common spatial organization across all timescales, the relaxed parameters proposed by Wang et al. implicate the possibility that individual regions oscillate at different frequencies. Future work should explore whether such relaxed model parameters that allow for frequency-sensitivity across space can improve the correspondence between EEG/MEG and fMRI FC.

To anticipate the studies covered in the following sections, empirical observations indeed show that a unifying connectome organization is qualitatively present in neurophysiological long-range FC of all oscillation frequencies, in line with the viewpoint of a timescale-invariant spatial organization. Furthermore, one might find it surprising that anatomical connectivity may explain a quite substantial proportion of the variance in a largely invariant neurophysiological FC organization (e.g., cf. *r*^2^ > 0.5; Finger et al., 2016). However, quantitative spectral differences (see sections 3b and 4) support an additional frequency-specific contribution in line with the viewpoint of a scale-sensitive FC organization.

We also note another difference across the two viewpoints with respect to their implications for an evoked versus intrinsic nature of FC (Raichle, 2009). When focusing on the functional specialization of each frequency band, one may expect that coupling would be primarily confined to time periods with particular processing demands directly associated with the given frequency band. Conversely, considering the observations of the above-described modeling approaches one would expect FC to occur in a largely ongoing and continuous manner. The neuroimaging field has recognized that the largest proportion of fMRI FC occurs in an intrinsic manner rather than as a reaction to external events and demands. Although direct rest-task comparison in neurophysiological connectomes are needed (see Box 1), we argue that the following sections are indicative of a similar scenario in neurophysiological FC in which a largely stable intrinsic spatial organization governs the majority of FC with minor yet cognitively consequential task-related changes.

Box 1. Future Directions We are likely to see the younger field of neurophysiological connectomes traverse equivalent research trends and advances previously observed and currently ongoing in the fMRI connectomics field. These trends include the following:

•  Identification of connectome changes in aging and neurodevelopment (Brookes et al., 2018).
•  Identification of connectome features affected by psychiatric and neurological conditions (Douw et al., 2019).
•  The study of individual differences, paralleling the respective fMRI-based developments (Finn et al., 2017; Kong et al., 2019; Mueller et al., 2013). The ability to identify monozygotic twins in MEG-based connectomes indicates feasibility to harness genetically driven individual differences (Demuru et al., 2017).
•  Time-varying FC dynamics and connectome state identification/clustering, extending on recent source space examples (instantaneous coactivation, Baker et al., 2014; and phase coupling, Vidaurre et al., 2018).
•  Comparisons across different mental states. It is known that fMRI-derived functional connectomes reconfigure only marginally during tasks compared with resting state (Cole et al., 2014; Gratton et al., 2018; Krienen et al., 2014), and the characterization of such subtle context-dependent reconfigurations is ongoing (Cohen & D’Esposito, 2016; Hearne et al., 2017). It has been reported that connectivity is spatially highly similar across levels of consciousness (Chu et al., 2012), and that topological graph properties are largely conserved across rest and a simple motor task for MEG in all canonical frequencies (Bassett et al., 2006), albeit in sensor space. It is important to perform such comparisons across mental states in source space whole-brain FC, especially since the possibility of a largely persistent intrinsic FC pattern is not commonly considered in neurophysiological investigations of task-related cognitive processes.

Additionally, direct cross-modal comparisons—including from concurrent multimodal recordings—are an especially promising avenue to address the following key challenges:

•  Dissociating the neurobiological scenarios that may give rise to the observed similarity of FC organization across timescales and data modalities, as discussed in the conclusions section. Integrating noninvasive modalities with intracranial electrophysiological recordings will be especially helpful to aid in this endeavor.
•  Identifying and quantifying the contribution of different factors leading to the remaining dissimilarity of FC organization across timescales and data modalities. Specifically, it is currently unclear to what degree such dissimilarity across frequency-specific neurophysiological connectomes and across neurophysiological and fMRI-derived connectomes is of biological nature or driven by data quality issues (cf. Box 2).

### 3b. Neurophysiology-Based Connectomes Are Spatially Linked to fMRI-based Connectomes

This section begins with evidence that an ICN organization governs both amplitude and phase coupling in various neurophysiological frequency bands. We then discuss direct comparisons of edgewise FC strength of the whole-brain connectome across EEG/MEG and fMRI. Note that most of the cross-modal comparisons discussed below (sections 3b, 3c, and 4) compare group-averaged rather than individual connectomes across modalities. In other words, they assess the similarity of the principal core of the connectome’s organization that is common to all subjects.

##### Presence of ICNs.

Initial evidence for the existence of a neurophysiological FC organization comparable to that observed in fMRI came from intracranial animal and human recordings (He et al., 2008; Nir et al., 2008; Shmuel & Leopold, 2008). Although intracranial recordings avoid source leakage issue, we limit our discussion of these studies as they typically lack whole-brain coverage (but see Betzel et al., 2019, for pooling over subjects). Intracranially recorded FC with spatial similarity to fMRI-derived ICNs spans all four orders of magnitude of neurophysiological signals and is observable in multiple FC metrics. Such similarity has been reported for cross-region correlations of the direct ECoG signal time courses (i.e., without spectral power or phase estimation) in the infraslow range (<0.5 Hz) (He et al., 2008) and in canonical frequency bands (Betzel et al., 2019), and for correlations of the band-limited amplitude envelope of high-*γ* (∼40–100 Hz) (Keller et al., 2013; Ko et al., 2013; Kucyi et al., 2018; Nir et al., 2008) and slower canonical frequency bands (Hacker et al., 2017).This similarity also holds for measures involving the phase of canonical oscillations (Betzel et al., 2019, Supplementary Material; Weaver et al., 2016).

For neurophysiological whole-brain connectomes typically inaccessible in intracranial recordings, we turn to MEG and EEG investigations. Although FC across the full MEG/EEG sensor space can be informative (e.g., Bassett et al., 2006; Betzel et al., 2012; Chu et al., 2012; Stam, 2004), we focus on source space connectomes to understand whole-brain FC organization across brain regions.

De Pasquale et al. used seed-based correlations of broadband MEG power (1–150 Hz) and reported evidence for the existence of the DMN, SM, dorsal attention network (DAT) (de Pasquale et al., 2010), visual (VIS), ventral attention (VAN), and language networks (de Pasquale et al., 2012). Hipp et al. investigated MEG seed-based power correlations at multiple logarithmically placed frequency bands. They observed auditory (AUD), VIS, SM, and DAT networks most dominantly carried by FC in the *α*/*β* range, and additionally the DMN as a set of regions with particularly high “hubness” (Hipp et al., 2012). Brookes et al. applied temporal ICA (rather than spatial ICA, as common in fMRI) to MEG amplitude envelopes of canonical frequency bands (*δ* through *γ*). They found multiple ICNs, including SM, VIS, fronto-parietal (FP), and cerebellar networks peaking in the *β*-band, and DMN in the *α*-band (Brookes et al., 2011). Similarly, studying broadband (4–30 Hz) instantaneous amplitude coactivation states, they observed recurring spatial FC patterns resembling DMN, VIS, and SM networks (cf. section Presence of “dynamic” connectivity reconfigurations below; Baker et al., 2014). Spatial ICA of EEG oscillation power over all canonical frequency bands followed by fMRI-informed clustering of the independent components confirms spatial similarity to fMRI-derived ICNs (Sockeel et al., 2016). While all above-described studies focused on power-based FC, MEG phase coupling shows a similar ICN-conform spatial distribution (Colclough et al., 2016; Hillebrand et al., 2012 albeit the authors of this study caution about methodological limitations).

##### Connection-wise correspondence to fMRI connectivity.

A conceptual advance in more recent MEG/EEG studies is the shift toward studying FC patterns across whole-brain parcellations. Once again, this advance parallels the progression occurring earlier in the fMRI literature, from a focus on characterizing ICNs to investigating whole-brain FC patterns. Using the same brain parcellation atlas across modalities enables quantitative comparison of their FC patterns, and we mention respective effect sizes where possible.

Hipp and Siegel (2015) performed connection-wise comparison of MEG FC (band-limited amplitude envelope correlations) and fMRI FC recorded in the same subjects. The correlation, that is, spatial correspondence, between the full MEG and fMRI FC matrices was significant but modest (*r* = 0.12 (0.38) prior to (respectively after) SNR normalization procedure using Spearman’s correction for attenuation). Although this spatial similarity to fMRI was present to some degree in all canonical frequency bands, the cross-modal similarity varied by connection and frequency. It is important to note that this study reported mean individual subject correlation. Contrarily, the studies discussed below report findings for group-averaged connectomes instead, which may explain their larger effect sizes.

Tewarie et al. (2016) directly addressed how FC in the numerous oscillation frequencies collectively contributes to fMRI-derived FC. They found that single-frequency band MEG-derived networks explain statistically significant but small variance in the whole-brain fMRI FC matrix (*r* up to 0.35 for amplitude coupling and *r* up to 0.24 for phase coupling). Importantly, prediction of the fMRI FC pattern substantially improved when jointly considering all canonical MEG frequencies, and further improvement was observed by including linear, nonlinear, and cross-frequency combinations of MEG FC values (*r* = 0.6 for amplitude coupling and *r* = 0.5 for phase coupling). This observation suggests that neurophysiological FC in different frequencies constitute not only common but also unique contributions to FC in fMRI, and that the cross-modal relationship contains nonlinear components. Interestingly, a comprehensive model that included both amplitude coupling and phase coupling was the best predictor of fMRI-derived FC (*r* = 0.73). This observation further suggests that beyond a common core, amplitude and phase coupling may be associated with unique and complimentary components of FC in fMRI.

##### Presence of “dynamic” connectivity reconfigurations.

Time-varying changes in FC deserve a dedicated discussion given the extensive current interest in dynamics in the field of fMRI connectomics (cf. section 2). Here, we discuss literature that assesses spontaneously occurring time-varying FC dynamics in EEG/MEG. Early studies of dynamics in sensor space EEG have indicated the presence of subsecond FC reconfigurations (Betzel et al., 2012; Chu et al., 2012), and have been extended to source-reconstructed connectomes: in MEG source space, hidden Markov models have been used to detect recurring spatial patterns of instantaneous coactivation of broadband (4–30 Hz) amplitude (Baker et al., 2014). As discussed above, this study found transient occurrences of several activity patterns, some of which resemble DMN, SM, and VIS ICNs observed in fMRI, but exhibiting much faster reconfigurations (∼100−200 ms). The same group extended the hidden Markov model approach to the *combination of* instantaneous amplitude and phase coupling (Vidaurre et al., 2018). This work confirmed rapid (∼50−100 ms) transient activation patterns in DMN, SM, and VIS networks constructed from broadband (1–45 Hz) amplitude. These coactivation patterns were accompanied by transient coherence across the same brain areas that showed coactivation. However, the DMN was reported to occur in the form of two independent patterns, a posterior subdivision operating primarily in the *δ*–*θ* range and an anterior subdivision employing the *α*-band.

Some parallels can be drawn between the observed MEG states and the states identified in fMRI-derived connectome dynamics. For example, some states are dominated by SM and sensory FC while other states are not (Allen et al., 2014; Vidaurre et al., 2017). Furthermore, a key dissociating factor between fMRI-based FC states is the connectivity profile of the DMN, and certain states similarly exhibit a posterior-anterior split of the DMN (e.g., Allen et al., 2014). It is unclear, however, whether one should expect fast-switching neurophysiological connectome states to directly correspond to or even “sum up” to the slower fMRI-derived states. Note that direct spatiotemporal correspondence and co-occurrence of time-varying FC changes across EEG/MEG, and fMRI can be assessed only when acquired concurrently (see section 3c on dynamics).

In summary, evidence for a reproducible ICN organization in neurophysiological signals is converging, with VIS, SM, and DMN among the most robustly reported networks. Furthermore, the reviewed whole-brain investigations collectively suggest that the intrinsic connectome organization known from fMRI is to some degree present in neurophysiological FC. This observation holds true for both phase- and amplitude-coupling measures. Although different frequencies may contribute to specific connections or networks to different degrees, the cross-modal correspondence of the whole-brain FC pattern is not confined to any particular oscillation frequency. Finally, dynamic neurophysiological FC investigations suggest that connectivity in the different ICNs occur in succession over shorter epochs. This dynamic pattern gives rise to the whole-brain connectome architecture when integrated over longer time periods.

In sum, oscillation-based networks are stable over long periods, and their organization is largely invariant to changing cognitive demands. Thus, the data suggest that phase coupling and amplitude coupling are primarily intrinsic processes. This conclusion extends the understating of neurophysiological FC beyond prevalent expectations of timescale sensitivity and mental-state dependence discussed in section 3a.

### 3c. Relation to fMRI Connectivity Recorded Concurrently

Research on the relationship of FC across fMRI and neurophysiological signals over the respective timescales benefits from concurrent measurements in two major ways. First, concurrent recordings ensure that recordings stem from the same mental state such as levels of vigilance that affect both measures. Second, concurrent recordings permit cross-modal comparison of time-varying changes in FC.

Studies recording EEG and hemodynamic signals concurrently have investigated the relationship between fluctuations of neurophysiological signal power with BOLD amplitude (Hiltunen et al., 2014; Laufs et al., 2003; Mantini et al., 2007; Sadaghiani et al., 2010b), neurophysiological signal power with BOLD FC (Allen et al., 2017; Chang et al., 2013a; Tagliazucchi et al., 2012b), and FC of neurophysiological signals (in sensor space) with BOLD amplitude (Jann et al., 2009; Sadaghiani et al., 2012). These observations span the full breadth of EEG timescales from infraslow fluctuations in direct current recordings (Hiltunen et al., 2014) to canonical oscillations (e.g., Allen et al., 2017; Mantini et al., 2007; Sadaghiani et al., 2012; Tagliazucchi et al., 2012b). Many of these studies have been covered elsewhere (for informative reviews see Keilholz, 2014; Schölvinck et al., 2013). Conversely, neurophysiological whole-brain connectomes, that is, source-reconstructed FC across whole-brain parcellations as detailed in Figure 1B and Figure 2, have only recently been extended to concurrent EEG-fMRI (Deligianni et al., 2014; Wirsich et al., 2017b). Note that as in section 3b, each of these connectome studies use the same atlas parcellation across the different data modalities.

##### Methodological considerations for concurrent recordings.

General considerations of concurrent EEG-fMRI, especially MRI-induced gradient and cardioballistic artifacts in EEG, are covered extensively elsewhere (e.g., Abreu et al., 2018). An additional consideration particularly important to connectomics is that different frequency bands might be affected by MRI-related artifacts to different degrees. Specifically, gradient artifacts are often stronger at high frequencies (Ritter et al., 2010). Furthermore, the helium pump responsible for cooling the superconductive MRI coil causes a vibration artifact in the EEG that leads at a scanner-specific peak frequency (in the *γ* range) and its harmonics, unless it can be turned off during data acquisition (Nierhaus et al., 2013). Another frequency-specific signal deterioration is the residual gradient artifact at a frequency defined by MRI excitation pulses per second, often remaining visible in the EEG spectrum after gradient artifact removal. Such frequency-specific disruptions of EEG oscillations and, consequently, EEG-derived FC must be considered when comparing EEG connectomes from different bands and may contribute to reduced effects in the high bands described below. Recent developments improving the coverage of electrodes on the scalp to up to 256 electrodes inside the scanner (Iannotti et al., 2015), and acceleration of fMRI volume acquisition (Uji et al., 2018), may help increase SNR of EEG and fMRI to strengthen the cross-modal relationship.

##### Cross-modal relation of “static” connectivity organization.

The few existing concurrent EEG-fMRI papers show convincing correspondence to fMRI FC irrespective of whether amplitude coupling or phase coupling is used. Deligianni et al. (2014) found significant spatial similarity across fMRI-derived and EEG-derived connectomes by using band-limited amplitude coupling. This similarity was stronger for lower frequency bands than for *β*- and *γ*-bands. Interestingly, when using a statistical prediction approach, prediction of fMRI-derived from EEG-derived connectomes performed substantially better than the other way around across all bands. The authors concluded that the EEG connectome irrespective of frequency band carries richer information than the fMRI connectome (at least at the spatial resolution of typical atlas parcellations, which may reduce fMRI resolution; cf. Box 3). In other words, the EEG connectome may carry additional information about neural FC not present in the fMRI-derived connectome, more so than the other way around.

Wirsich et al. (2017b) used EEG phase coupling (imaginary part of the coherency) and similarly observed a similarity between EEG and fMRI connectomes. The spatial correspondence was *r* > 0.3 for all bands except *γ* (*r* = 0.16). Both Wirsich et al. (2017b) and Deligianni et al. (2016) further assessed the relation to structural connectivity as discussed in section 4. Of special importance, the only concurrent *intracranial* EEG and fMRI study on FC existing to date has delivered evidence for a spatial correspondence of FC strength across modalities without requiring source reconstruction. Specifically, Ridley et al. (2017) investigated FC in ECoG and depth electrodes based on amplitude envelopes. In normal (nonepileptic) regions, they found small but significant spatial correspondence to concurrent fMRI FC strength for all canonical bands (*δr* = 0.19, gradually decreasing through *γr* = 0.05) and for broadband EEG (*r* = 0.09).

##### Cross-modal relation of ‘dynamic’ connectivity reconfigurations.

Evidence for electrophysiological correlates of fMRI-based FC dynamics from concurrent multimodal studies has been informatively reviewed elsewhere (Keilholz, 2014; Thompson, 2017). For instance, neurophysiological oscillations recorded invasively in the rat show interhemispheric FC across homologous somatosensory areas that cofluctuate with concurrent fMRI-derived dFC across the same regions (Pan et al., 2011; Thompson et al., 2013). In the concurrent human intracranial EEG and fMRI study mentioned above (Ridley et al., 2017), region pairs with higher variability in fMRI-derived FC also had higher variability in EEG-derived FC of *α*-, *β*-, and *γ*-bands (where variability was measured as standard deviation of FC over time). Unfortunately, the limited spatial coverage of such invasive electrophysiology studies does not inform about the cross-modal correspondence of dynamic changes in the functional connectome’s whole-brain spatial topography.

Although scalp EEG and fMRI provide whole-brain coverage, the vast majority of existing time-varying investigations of concurrent EEG-fMRI do not assess EEG source space connectivity. Several studies have instead focused on EEG power correlates of fMRI-derived FC dynamics. A prominent example is the study of EEG microstates, quasi-stable topographies of momentary scalp distributions (in sensor space) typically extracted from broadband global field power. Several such microstates have been identified whose occurrence coincides with activa tion in well-known ICNs in concurrent fMRI (Britz et al., 2010; Musso et al., 2010; Van De Ville et al., 2010). Interestingly the puzzling correlation between brief microstates (∼ 50 − 100 ms) and the slow dynamics of intrinsic BOLD signal fluctuations (∼ 5 − 10 s) may be explained by scale-free, self-similar dynamics of microstates that span over several scales from 256 ms to 16 s (Van De Ville et al., 2010). This observation bridges across fast neural dynamics and slowly fluctuating ICN organization.

Beyond microstates, periods of high and low EEG power in gross electrode groups have been associated with various fMRI FC features. Examples of these features include DMN-DAT anticorrelation (Chang et al., 2013a) or the average path length in the fMRI graph (Tagliazucchi et al., 2012b). The fMRI FC to EEG power relationship can substantially differ across frequencies. For example, the latter study (Tagliazucchi et al., 2012b) found that fluctuations in *α*- and *β*-power co-occurred with widespread *decrease* in fMRI-derived FC, while *γ*-power was associated with increase in long-range fMRI FC. Extending below the typically recorded EEG frequency range, Keinänen et al. (2018) found that infraslow (<0.1 Hz) EEG and BOLD signal fluctuations are more strongly correlated during periods of high fMRI FC in the DMN. Another study decomposed EEG spectral power over all sensors into spatiotemporal activity patterns with different spectral fingerprints. The time course of three such patterns temporally correlated with sliding window fMRI FC dynamics across specific ICN pairs (Lamoš et al., 2018). In line with an aforementioned study (Tagliazucchi et al., 2012b), a spatiotemporal EEG pattern with high *α*- and *β*-power dominated when between-ICN fMRI FC was low (Lamoš et al., 2018). Following the reverse analysis direction, Allen et al. (2017) first dissociated dynamically recurring fMRI connectome states by applying a clustering algorithm to sliding window fMRI FC. They found that the EEG power spectrum co-occurring with these distinct fMRI connectome states differed from one another in certain electrode groups.

Beyond these studies on EEG power correlates of fMRI FC dynamics, a recent study assessed EEG FC albeit in sensor space. The study identified recurring EEG connectivity states (4–30 Hz broadband amplitude envelope correlations) by using hidden Markov models, and found that fMRI coactivation patterns co-occurring with these EEG states resembled traditional ICNs (Hunyadi et al., 2018).

To summarize section 3c, the few existing studies comparing concurrently recorded fMRI connectomes and source-reconstructed EEG connectomes have established a cross-modal similarity of static FC organization. Concurrently recorded EEG and fMRI are particularly useful to understand the cross-modal relationship of time-varying dynamics, and several dynamic investigations have provided insights into EEG power and EEG sensor-level FC. To allow for a direct comparison of whole-brain connectome reconfigurations across modalities, investigations of source-reconstructed concurrent EEG will be required in the future.

To conclude, temporal convergence of spontaneous time-varying changes across concurrent neurophysiological and fMRI measures supports a neural origin of fMRI-derived connectomes (Schölvinck et al., 2013). This implication is especially critical in light of the susceptibility of fMRI-derived FC dynamics to contamination from noise and sampling error (Keilholz, 2014). Although this methodological conclusion is important, another implication is more profound in our opinion. Specifically, the cross-modal static and dynamic convergence provides support for a neural FC organization that crosses timescales, as further discussed in section 5.

## 4. THE RELATIONSHIP ACROSS FUNCTIONAL AND STRUCTURAL CONNECTOMES

In the following, we discuss to what degree fMRI-based and neurophysiological connectomes can be predicted from the underlying neural fiber tracts derived from dMRI-based tractography. We close with trimodal studies of dMRI-, fMRI-, and neurophysiology-derived connectomes that allow researchers to directly compare the structure-function relationship of fMRI and neurophysiological modalities.

### 4a. The fMRI-based Connectome Is Linked to the Structural Connectome

Moderate but significant correlation (*r* ∼ 0.3) has been reported between connection strength of the structural and the fMRI-derived connectomes (Honey et al., 2009; Skudlarski et al., 2008). Furthermore, simulations discussed in section 3a suggest that structural connectivity mechanistically contributes to fMRI-derived FC organization (Deco et al., 2011). As functional connections can be realized without a direct structural connection (Damoiseaux & Greicius, 2009), several studies have focused on better modeling the function-structure relationship, for example, by using network communication theory (Goñi et al., 2014), generative models (Betzel et al., 2016), or partial least squares (Mišić et al., 2016).

When modeling BOLD FC from the anatomical structure, the model predicts a closer cross-modal relationship when averaging simulated FC across longer time periods (Honey et al., 2007). However, the inherently flexible nature of cognition and the observation of FC dynamics (cf. section 2) lead to the question of when/how the functional connectome undergoes flexible excursions away from the structural core that provides its stable foundation. By applying a point process analysis that can identify discrete spatiotemporal events in fMRI (Tagliazucchi et al., 2012a), it has been shown that networks of transient spatiotemporal FC selectively propagate along structural connections in a complex wave-like pattern (Griffa et al., 2017). Fukushima et al. found that the fMRI-derived connectome is most similar to the structural connectome when the former is in a highly integrated state. In other words, increased modular segregation of the functional connectome reflects flexibility away from the structural connectome (Fukushima et al., 2017). Empirically observed dynamics of fMRI-derived FC can be simulated by combining structural connectivity with stochastic processes (Hansen et al., 2015). However, how FC dynamics are shaped by structural connectivity varies over network nodes (Shen et al., 2015), and an exact explanation of this relationship is still outstanding.

### 4b. Neurophysiology-based Connectomes Are Linked to the Structural Connectome

A close link to anatomical connectivity would provide convincing evidence for the true nature of source space neurophysiological connectomes, despite their methodological limitations (cf. Box 2). Although dMRI-derived tractography has its own methodological issues (Maier-Hein et al., 2017), spatial convergence across such reconstructed white-matter connectivity and neurophysiological connectomes would speak to reliability of both modalities. Once again, this conceptual evolution parallels that in fMRI in which the existence of intrinsic FC was similarly validated by its spatial relationship to structural connectivity (Honey et al., 2009; Skudlarski et al., 2008; van den Heuvel et al., 2009).

Furthermore, it is important to emphasize the relation of neurophysiological FC to the stable anatomical structure from a conceptual point of view, since neurophysiological FC has traditionally been thought of as rapidly forming and dissolving circuits, depending on cognitive demands (Kahana, 2006; Singer, 1999; Varela et al., 2001). Even in relatively speaking more recent conceptual frameworks on the role of oscillation-based FC in cognition, an intrinsic and largely persistent whole-brain spatial organization is not central (Buzsáki & Watson, 2012; Gratton, 2018) or only starting to be integrated (Singer, 2013).

Specifically, EEG source-reconstructed FC in all canonical frequency bands (*δ* through *γ*) is higher between nodes with direct and indirect dMRI-derived connections compared with those without structural connections (Chu et al., 2015, in epileptic children). This structure-function relationship persisted even after accounting for the contribution of spatial proximity to connectivity strength in both modalities, and was observed for both amplitude correlations and phase coupling. To mechanistically establish a contribution of structural to neurophysiological connectome organization, Cabral et al. set up a simple model (Kuramoto, two parameters only) of simulated phase-coupled oscillators based on real dMRI-derived FC. As discussed in section 3a, the simulated neurophysiological data spontaneously exhibited amplitude coupling. This coupling showed considerable spatial similarity to real MEG amplitude coupling in various frequency bands (*r* = 0.41 for the optimal model parameters) (Cabral et al., 2014). A study of EEG a-band phase coupling confirmed a close structure-function relationship (*r* = 0.48) (Finger et al., 2016). Compared with using structural connectivity as a direct predictor, the association substantially increased (*r* = 0.74) when dMRI connectivity was entered into a (Kuramoto) model of phase-coupled oscillators to simulate EEG FC.

Box 2. Methodological considerations: fMRI connectomes **Dealing with noise.**

Addressing nonneural sources of noise is crucial for fMRI-based FC. Such noise is considerably more likely to cause false positives/distortions in fMRI-based intrinsic FC than task-related fMRI activation, since the former lacks experimentally controlled timing of events of interest. By its very nature, the BOLD signal is susceptible to respiration and heart rate (Birn et al., 2006; Chang et al., 2013b). To reduce this impact, regression of peripheral physiological recordings (Glover et al., 2000) and data-driven decomposition approaches (e.g., Behzadi et al., 2007) are being used. For a more detailed review see Power et al. (2017). Subsecond sampling rates of more recent accelerated fMRI sequences are expected to reduce aliasing of physiological noise and improve cleaning methods. Similarly, head motion can lead to systematic and widespread, but not uniformly distributed, changes in fMRI-based FC (Van Dijk et al., 2012). Although remaining a key concern, the fidelity of fMRI-based FC can improve considerably through removal of high-motion subjects and volumes or interpolation of such volumes, regression of estimated head motion or global signal shifts (but see Bright et al., 2017, for methodoloigcal challenges of nuisance regression), and data-driven decomposition (Parkes et al., 2018; Siegel et al., 2017).

**Parcellation schemes.**

Although possible, fMRI voxel-wise FC estimation is computationally expensive and may be conceptually suboptimal. Instead, the goal is typically to assess FC between regions that are functionally homogeneous. Homogeneity can be defined in numerous ways, however, including uniformity of cytoarchitecture, task-evoked functional response, or FC at either the group or individual subject level^1^ (Arslan et al., 2017). It is worth noting that for comparisons to other data modalities, atlases are often inevitably confined to delineation of gross anatomical landmarks (cf. section 3a). Although dependence of findings on the chosen parcellation scheme is inevitable, researchers can demonstrate the robustness of their findings by replication in a second scheme.

**Connectivity measures.**

Regarding measures of temporal dependency of fMRI timeseries, Pearson’s correlation is by far the most common approach. However, fMRI-based FC can be conceptualized in various other ways, including partial correlations (Marrelec et al., 2006), measures of nonlinear dependencies (Hlinkaa et al., 2011), or effective connectivity (Frässle et al., 2018; Stephan & Friston, 2010). Coupling of the phase of BOLD fluctuations has also been used (e.g., Sun et al., 2004), although such fluctuations lack the oscillatory nature that is a hallmark of ongoing neurophysiological activity. Additional measures have been proposed specifically for time-varying changes in fMRI FC (e.g., Shine et al., 2016). Although the choice of FC measure depends on the question at hand, Pearson’s correlation has proven to be an intuitively interpretable and informative metric in the broadest set of fMRI FC studies over the past decade.

**Mental states.**

Intrinsic FC is most commonly recorded in task-free resting state. However, subject instructions differ considerably across resting-state scans. For example, subjects may be asked to rest with eyes closed or fixate on a central visual marker. Similarly, they may or may not receive instructions to control their flow of thought (e.g., avoid fixating on any particular thought). Although some aspects of static fMRI FC (particularly ICNs) are extremely robust to these choices, the choices may be of substantial consequence for cross-modal comparisons (e.g., cf. sensitivity of *α*-band neurophysiological FC to eyes open/closed condition; Gómez-Ramírez et al., 2017). More recently, it has been suggested that dissociating individuals based on their functional connectome—useful for understanding interindividual differences in behavior and clinical conditions/outcomes—can be improved by constraining functional connectome states through stimulation with an identical continuous movie or story for all subjects (Finn et al., 2017). Some investigations even apply connectome approaches to fMRI data from more traditional task settings containing discrete events in the same way typically performed for continuous states (e.g., Bassett et al., 2011). This may be especially useful for understanding distributed FC reconfigurations during performance of cognitive tasks (see 2a section on dynamics, e.g., Shine et al., 2016).

The core conceptual difficulty of applying connectomics to task data is that the stimuli will increase temporal dependency across concurrently activated regions without necessarily reflecting information exchange or FC across those regions (Sadaghiani & Kleinschmidt, 2013). On the other hand, the presence of any task or stimulation has only minimal impact, at least on the time-averaged (i.e., static) fMRI-derived connectome (Cole et al., 2014; Gratton et al., 2018; Krienen et al., 2014), and task settings can be conceptualized as providing (minor) modulation to the connectome’s dynamic trajectory (Bolt et al., 2017).

**Temporal scale.**

In the context of this cross-modal review it is important to recall the low-pass characteristics of the BOLD signal; by nature, BOLD imaging is limited to the slow speed of neural activity–induced change in the concentration of deoxy-hemoglobin irrespective of speed of data acquisition. Therefore, fMRI-based FC can only measure the temporal relationship of very slow BOLD signal fluctuations, cutting off little above the range of the infraslow frequencies. However, distant neural populations exchange information at speeds of 10s of milliseconds and employ cross-region coupling of oscillatory activity in frequencies up to ∼100 Hz (cf. section 3a). Only the slower fluctuations in the regional amplitude (envelope) of such fast neural oscillations would result in fluctuations of metabolic demands measurable in the BOLD signal. Consequently, many studies aiming at understanding the neurophysiological counterpart of fMRI-based FC in multimodal recordings use amplitude coupling of neurophysiological signals (e.g., Nir et al., 2008; Thompson et al., 2013). However, neurophysiological FC can be conceptualized in terms of different mechanisms (cf. section 3a and Figure 3). Thus, how BOLD signal–derived FC relates to the FC of the underlying neurophysiological processes remains a complex issue (Schölvinck et al., 2013; Thompson, 2017).

### 4c. Trimodal Comparisons

A few studies have assessed the connection-wise relationship across dMRI, fMRI, and neurophysiological connectomes. Amplitude coupling in MEG was spatially well correlated with dMRI-derived connectivity for *θ* through *γ* bands (*r*_*Spearman*_ = 0.33 − 0.45; weaker for *δ*: *r*_*Spearman*_ = 0.14), outperforming the association across dMRI and fMRI (*r*_*Spearman*_ = 0.28) (Garcés et al., 2016). These dMRI-MEG and dMRI-fMRI associations weakened but persisted after accounting for contribution of physical distance. A dMRI model exploration found that Euclidian distance combined with a structural hub-to-hub connectivity measure in a temporo-parietal network is linked to *α*-band MEG phase coupling at $r_{adjusted}^{2}=0.12$, compared with $r_{adjusted}^{2}=0.33$ for fMRI FC (nonoverlapping populations for functional and structural data; Tewarie et al., 2014). Their follow-up study additionally confirmed a role of multinode (indirect) structural connections in predicting FC, and this dMRI-based prediction was more accurate for *α*-band MEG FC than it was for fMRI (Meier et al., 2016).

Three of the trimodal studies recorded EEG and fMRI concurrently (cf. section 3c). Schirner et al. (2018) showed that EEG power injection improves the fit of empirical resting-state fMRI FC to simulated data modeled from dMRI connectivity. Deligianni et al. (2016) investigated which connections of EEG-derived (amplitude coupling) and fMRI-derived connectomes predict various structural indices of anatomical connections. They observed that all EEG bands and fMRI mapped onto structural indices in a similar set of connections. Wirsich et al. (2017b) found that a measure of structural path length (weighted by the number of tracks between a given region-pair) is linked to EEG phase coupling with *r* = 0.34 (*γ*) to *r* = 0.54 (*β*), compared with *r* = 0.41 for the relation to fMRI-derived FC.

In summary, the partial spatial correspondence between neurophysiological and structural connectivity organization, albeit often weak in effect size, is reassuring. This is especially important for EEG since spatial localizability of neural activity is more limited than for MEG (although ameliorated by high-density EEG recording; Marquetand et al., 2019). The ability to study the whole-brain connectome in EEG is especially useful for concurrent acquisition with fMRI currently not possible for MEG (see section 3c), adding to EEG’s advantage in terms of low cost.

### 4d. Dissimilarities Across fMRI-based and Neurophysiological Connectomes and the Role of Structural Connectivity Therein

What causes the correspondence between neurophysiological and fMRI-derived connectomes (section 3) to be imperfect? It is generally difficult to determine which part of neurophysiological FC organization is genuinely different from fMRI FC and which part is due to noise in either modality (cf. Box 2 and Box 3). One approach to address this issue is to compare both FC modalities to structural (dMRI) connectivity as “ground truth.” In this approach, any FC information that explains dMRI is considered true FC as opposed to noise. Despite dMRI’s own methodological issues, this approach has proven informative.

Box3. Methodological considerations: Neurophysiological connectomes The study of whole-brain connectomes in MEG/EEG entails important methodological considerations that have been covered in informative reviews (e.g., O’Neill et al., 2017) and extended to network neuroscience (graph theory) approaches (Hassan & Wendling, 2018). We therefore only briefly touch on core methodological issues.

**Source reconstruction.**

First and foremost, MEG- and EEG-based whole-brain connectomics require source projection, and empirical comparisons show that the spatial topology of FC differs heavily between source and sensor space (Lai et al., 2018). In EEG, electrical signals spread over the head because of volume conductance, although high-density EEG recording may increase the reliability of EEG FC to levels similar to MEG (Marquetand et al., 2019). For both EEG and MEG FC, source localization is required because the mapping of EEG/MEG sensor data to brain regions is not linear, and several sensors can detect the activity of the same neuronal source. Consequently, source projection is an ill-posed problem with many possible solutions, that is, numerous possible source constellations for relatively few sensors. Because of spatial source reconstruction methods being subject to these spatial uncertainties, the smearing of the source signal over a relatively large brain volume can cause source leakage in the reconstructed source space (Farahibozorg et al., 2018).

This issue is commonly addressed by excluding any relationship at zero lag between two signal time courses, as leakage is assumed to propagate instantaneously (Palva & Palva, 2012). Common approaches include removing the linear dependencies by orthogonalization of the signal (Brookes et al., 2011), or only considering FC that does not occur at zero phase lag, for example, by using the imaginary part of the coherency (Nolte et al., 2004), the imaginary part of the phase-locking value (Sadaghiani et al., 2012), or an index of asymmetry of the phase-difference distribution (Stam et al., 2007). Unfortunately, this conservative approach comes at the cost of removing real zero lag long-range FC whose existence (e.g., Gray et al., 1989; Rodriguez et al., 1999; Roelfsema et al., 1997) and contribution to the whole-brain connectome (e.g., Finger et al., 2016) are supported empirically and theoretically (Viriyopase et al., 2012).

For estimation of region-wise time courses, densely positioned sources can be estimated and then averaged within parcellation regions (e.g., Deligianni et al., 2014; Wirsich et al., 2017b) with optional weighting by the distance to the center of mass of each parcellation region (Brookes et al., 2016; Tewarie et al., 2016). Other options include singular-value decomposition (Colclough et al., 2016; Rubega et al., 2019), or restricting the regions to sparsely and homogenously distributed solution points (e.g., Hipp & Siegel, 2015).

It should also be noted that the parcellation atlases used in MEG/EEG studies are comparable to those applied to fMRI but with two limitations. First, subcortical areas are typically not considered estimable for surface recordings. Second, the spatial resolution or number of parcels is limited by the number of recorded sensors. When averaging the source activity to an anatomical atlas, the above-described spatial uncertainty of the true neuronal source can lead to mixing one source into several parcellation regions. This issue can be ameliorated by minimizing the cross-talk between regions (Farahibozorg et al., 2018). Parcellating the brain according to the cross-talk or by bundling close nodes together can further minimize spurious FC (Palva et al., 2018). The latter issue also means that studies performing connection-wise comparisons across neurophysiological and MRI-based connectivity may be limited to relatively low-resolution parcellations for MRI as well.

**Connectivity measures.**

Another core methodological issue of importance for cross-modal comparisons concerns the definition of FC in neurophysiological signals (Schölvinck et al., 2013). fMRI measures neural activity fluctuations (and consequently FC), most notably in the infraslow (<0.1 Hz) range because of the low-pass characteristics of the hemodynamic signal. Contrarily, neurophysiological methods have real-time resolution and can cover signal variations over ∼4 orders of magnitude from infraslow fluctuations (if permitted by appropriate recording hardware) to the range of “canonical” band-specific oscillations (*δ* through high *γ*; 1 through ∼100 Hz). Consequently, whereas the vast majority of fMRI-based connectome studies use some measure of statistical dependency of signal amplitudes (commonly Pearson’s correlation), neurophysiology-based FC can be derived both on the basis of amplitude coupling as well as phase coupling (for more complex cross-frequency dependencies see Discussion section). As Figure 3 demonstrates, it is plausible to assume that the two types of measures capture mechanistically different neurophysiological FC phenomena.

Indeed, on this theoretical basis it has been proposed that amplitude coupling regulates the (co)activation of brain regions, while phase coupling regulates the integration and flow of cognitive contents (Engel et al., 2013). Unfortunately, neurophysiological FC in cognitive tasks is primarily studied using phase coupling (e.g., Hirvonen et al., 2018) (for an exception see Luckhoo et al., 2012). Contrarily, the neurophysiological FC investigations at resting state use both phase-coupling and amplitude-coupling measures, with increasingly high prevalence of the latter (see sections 3b–3d; e.g., Brookes et al., 2011; de Pasquale et al., 2012; Hipp et al., 2012). The difference in the respectively preferred FC metric has resulted in some disconnection between the task-based and resting-state neurophysiological literatures. In task-free resting-state MEG, high similarity (*r* >= 0.7) was observed across the group-averaged FC matrices from various amplitude- and phase-coupling measures (albeit amplitude coupling yielded higher intersubject reliability) (Colclough et al., 2016). The spatial similarity of amplitude and phase coupling–based neurophysiological connectomes is further supported indirectly by the similarity of both to fMRI-based connectomes (see sections 3b and 3c).

For example, neurophysiological connectomes may reflect structural connectivity slightly better than fMRI connectomes do (at least in relatively low-resolution parcellations typically used for multimodal studies; Garcés et al., 2016; Wirsich et al., 2017b). One likely contributor to this observation is that in the absence of direct structural connections, EEG-based FC is small (Chu et al., 2015), paralleling dMRI connectivity. Conversely, fMRI-based FC may be strong even in the absence of direct structural connections (Damoiseaux & Greicius, 2009), which may weaken dMRI-fMRI associations compared with dMRI-EEG. Indeed, in their trimodal studies, both Wirsich et al. (2017b) and (Deligianni et al., 2016) report converging evidence that homologous connections are stronger in fMRI, whereas EEG is more strongly characterized by intrahemispheric connections. This convergence was observed despite very different EEG FC metrics used in the two studies (phase vs. amplitude coupling, and correcting for vs. not excluding zero lag FC).

Wirsich et al. (2017b) also demonstrated some degree of frequency specificity in the above-described complimentary information that EEG contributes to the structure-function relationship. While *δ* contributed globally to predicting structural connectivity, *γ* contributed local information in the visual network. This observation suggests a common core shared across EEG, fMRI, and dMRI connectivity, with additional smaller modulations on different timescales. Beyond insights from comparisons to structure, causes of the divergence between neurophysiologcial and fMRI-derived connectomes remain open for future investigations (cf. Box 1).

To conclude section 4, the structural backbone mechanistically contributes to both fMRI-derived and neurophysiological connectomes in various frequency bands (Cabral et al., 2014; Honey et al., 2009), despite some modality- and frequency-specific connectivity differences. Thus, a common structural basis provides a foundation for spatial similarity across fMRI-derived and neurophysiological connectomes.

## CONCLUSIONS

Based on fMRI observations, it has been widely accepted that infraslow neural activity and its cross-region temporal dependencies are governed by a stable spatial organization that is intrinsic in nature, that is, largely independent of mental states or external tasks (Petersen & Sporns, 2015). The reviewed literature suggests that this intrinsic functional architecture is not unique to the timescales of the hemodynamic signal. Rather, intrinsic FC is present in neurophysiological data independent of particular cognitive processes and irrespective of temporal scale (frequency band) or coupling mode (phase and amplitude coupling). Neurophysiological FC further shows small to moderate but significant spatial similarity to the FC organization commonly observed in fMRI. We focus the following discussion on neurobiological scenarios that can explain these similarities.

Regarding the relationship across neurophysiological and hemodynamic FC patterns, the reviewed work is in line with the notion that numerous neurophysiological FC phenomena (phase and amplitude coupling in various bands) contribute to a unitary hemodynamic signal (Schölvinck et al., 2013). As discussed in section 3a, the more intriguing question is how a fairly stable and reproducible FC pattern, or intrinsic “cognitive architecture” (Petersen & Sporns, 2015), can to some degree be shared among this large range of neurophysiological FC measures. Several scenarios are conceivable.

On the one hand, it is important to remember that spectral power at a given frequency does not necessarily imply existence of oscillations at that center frequency. For example, bursts of activity with sharp on- and offsets (Gratton, 2018) will have a broad spectral fingerprint, that is, power across frequencies covering all canonical oscillation bands. On the other hand, comparisons in the time domain have been able to dissociate broadband events such as bursts from continuous oscillations, and one such investigation speaks against the broadband account (Deligianni et al., 2014). This study demonstrated low correlation between the different bands’ region-wise time series. This observation suggests that the whole-brain EEG connectomes in the different canonical frequency bands are not driven by broadband signal changes. Rather, EEG signals at different frequencies seem to operate within the same networks (Deligianni et al., 2014).

Multiple scenarios are in line with the possibility that a given pair of regions may indeed connect to each other using the full breadth of oscillatory frequencies. Since intrinsic FC is often derived over extended periods (in the order of minutes), different neurophysiological FC phenomena could dynamically emerge and fade in succession. The low correlation between individual bands’ FC time series discussed above speaks to a contribution of this scenario (Deligianni et al., 2014). This possibility is further supported by observation of frequency specificity of dynamic neurophysiological FC states (cf. dynamics section in 3b; Vidaurre et al., 2018).

Additionally, FC in different frequency bands could occur in different neural populations within the same regions. A prominent example of this possibility suggests that *γ* oscillations primarily occur in feed-forward connections originating in superficial cortical layers, while *α*- and/or *β*-oscillations occur in feedback connections originating in deep layers, supported by the observation that at least in sensory cortices *γ* and *α*/*β* are expressed to different degrees in different layers (Fries, 2015; Scheeringa & Fries, 2019; Siegel et al., 2012).

Yet another possibility is that different frequency bands reflect temporally interdependent activity patterns across and within neural populations. Periodic interdependencies across frequencies are referred to as cross-frequency coupling (CFC; Canolty & Knight, 2010). Both at rest and during task, this scenario finds support in empirical observations of phase-amplitude CFC and phase-phase CFC binding (Siebenhuehner et al., 2019; Siebenhuehner et al., 2016). In phase-amplitude CFC the phase of a relatively slow frequency modulates the amplitude of faster frequencies, whereas phase-phase CFC binds the phases across different frequencies. The likely most common example of CFC is neural activity oscillating in the *γ* range within time segments rhythmically intermitted at *α*-, *θ*-, or *δ*-frequency. This phenomenon is observed in representations of items in working memory (Axmacher et al., 2010; Siegel et al., 2009), rhythmic sampling of sensory information (Lakatos et al., 2005), and their attentional modulation (Jensen et al., 2014; Lakatos et al., 2008).

An interesting implication of a hierarchy of phase-amplitude CFC is that it may explain the puzzling observation of a comparable spatial functional connectome pattern not only across temporal scales but also across FC measures, that is, both phase and amplitude coupling. Importantly, CFC may hold across a series of frequencies in a hierarchical manner (Lakatos et al., 2005). Mechanistically, such a hierarchy could allow large-scale and distributed processes in relatively slower frequencies to modulate more local and faster processes (Canolty & Knight, 2010), with the consequence that even fast processes can show coupling across large distances. As discussed in section 3a this multiscale structure is backed by brain modeling approaches showing a common scale-free pattern supported by the anatomical backbone of the brain. To consider an analogy, rather than thinking of FC in different frequency bands as a means of protecting parallel and largely independent “conversations” from each other, they would correspond to parallel melody lines of a polyphonic choir that hold an overarching temporal relationship to each other for a holistic outcome.

An additional observation indicates that hierarchical CFC “nesting” may hold across the full frequency spectrum from infraslow to high *γ*. Specifically, the power density of neurophysiological data is inversely proportional to oscillation frequency. This *1/frequency* power relationship may imply that perturbations occurring at slow frequencies cause a cascade of energy dissipation at higher frequencies. Consequently, widespread slow oscillations modulate faster and more local events (Buzsáki & Draguhn, 2004; Zhigalov et al., 2017).

To conclude, rapid temporal switches across FC in different frequency bands within the same spatial organization (shaped by anatomical structure), spatial overlap of neural populations with different frequency preferences, and CFC are plausible explanations for an intrinsic FC organization that comprehensively covers mental states, timescales, and FC measures.

## AUTHOR CONTRIBUTIONS

Sepideh Sadaghiani: Conceptualization; Writing - Original Draft; Writing - Review & Editing. Jonathan Wirsich: Writing - Original Draft; Writing - Review & Editing.

## FUNDING INFORMATION

Sepideh Sadaghiani, National Institute of Mental Health (http://dx.doi.org/10.13039/100000025), Award ID: 1R01MH116226-01A1.

## Notes

1 For a diverse list of current atlases see https://www.lead-dbs.org/helpsupport/knowledge-base/atlasesresources/cortical-atlas-parcellations-mni-space/.

## TECHNICAL TERMS

Functional connectivity (FC): At the systems level denoting the temporal dependency of signal time courses (e.g., from fMRI, EEG, or MEG) measured from distributed brain regions.
Connectome: A whole-brain map of structural or functional neural connectivity. At the systems level, connections are typically established among brain regions, e.g., of a brain atlas.
Canonical oscillations: Rhythmic variations of neural population activity observable in specific frequency bands thought to represent different neural processes, including *δ* (∼1–3Hz), *θ* (∼4–7Hz), *α* (∼8–13Hz), *β* (∼15–25Hz), and *γ* (>30Hz).
Default mode network (DMN): A network or ICN of distributed brain areas that show increased activation as external cognitive demands diminish.
Intrinsic connectivity networks (ICNs): Networks that spontaneously exhibit temporal dependency among neural activity time courses of their distributed regions. Regions of a given ICN also co-activate in response to the same cognitive demands.
Sources: Plausible neural generators in the brain hypothesized to cause the signals observed at EEG or MEG sensors over the scalp.
Source leakage: Smearing of the source signal over a relatively large brain volume in the reconstructed source space (cf. Box 3).
Amplitude coupling: The similarity of amplitude envelopes of band-limited oscillations, i.e., how the change in strength of a particular oscillation is coupled across two locations. Commonly quantified as correlation of envelopes (from Hilbert transform or power of the spectrum at specific frequency).
Phase coupling: The consistency of phase lag between two oscillatory time courses as quantified by various measures, e.g.,(imaginary) coherence, phase locking value, and phase lag index.
Structural (or anatomical) connectivity: A representation of the physical white-matter connections between distant brain regions usually derived from diffusion MRI.
